# Spontaneous Intramuscular Hemorrhage in Anti-melanoma Differentiation-associated Gene 5 Antibody-positive Dermatomyositis

**DOI:** 10.31662/jmaj.2024-0412

**Published:** 2025-02-28

**Authors:** Tomoyuki Mutoh, Hidetoshi Mitsui, Hiroshi Fujii

**Affiliations:** 1 Department of Rheumatology, Osaki Citizen Hospital, Osaki, Japan; 2 Department of Dermatology, Osaki Citizen Hospital, Osaki, Japan; 3 Department of Rheumatology, Tohoku University Hospital, Sendai, Japan; 4 Department of Clinical Immunology and Rheumatology, Tohoku University Graduate School of Medicine, Sendai, Japan

**Keywords:** spontaneous intramuscular haemorrhage, dermatomyositis, autoantibody, MDA5, Ro52, rapidly progressive-interstitial lung disease, factor XIII

## Abstract

Anti-melanoma differentiation-associated gene 5 (MDA5) antibody-positive dermatomyositis (DM) is characterized by mild or absent muscle involvement and unique skin lesions such as cutaneous ulceration and palmar papules, commonly associated with rapidly progressive interstitial lung disease (RP-ILD), causing fatal outcomes. Spontaneous intramuscular hemorrhage (SIH) is an extremely rare but severe complication that remains under-recognized in DM. Here, we report a case of multiple SIH in a 72-year-old Japanese woman with anti-MDA5 antibody-positive DM and RP-ILD. The patient initially presented with fever, fatigue, and abnormal liver function, leading to a provisional diagnosis of autoimmune hepatitis. Following a 3-week moderate-dose prednisolone treatment, bilateral thigh hematomas suddenly developed without trauma or anticoagulant therapy. Laboratory findings revealed elevated creatine kinase and ferritin levels, reduced factor XIII (F13) activity, and anemia. Computed tomography (CT) imaging showed hematomas in multiple muscles and ILD. Although high-dose prednisolone administration gradually ameliorated the intramuscular hemorrhage, skin manifestations indicative of DM and dyspnea on exertion emerged after tapering prednisolone. Exacerbation of ILD was observed on CT imaging. Comprehensive analysis detected anti-MDA5 and anti-Ro52 antibodies without anti-F13 autoantibody, eventually leading to the diagnosis of anti-MDA5 antibody-positive DM with SIH and RP-ILD. Despite aggressive immunosuppressants, she died of RP-ILD-related respiratory failure. This case highlights the importance of considering DM as a differential diagnosis and investigating cutaneous manifestations indicative of DM in cases where the SIH etiology is unclear. Evaluation of myositis-specific and myositis-associated autoantibodies is crucial to ensure adequate diagnosis when SIH associated with DM is highly suspected.

## Introduction

Anti-melanoma differentiation-associated gene 5 (MDA5) antibody-positive dermatomyositis (DM) typically presents mild or absent muscle involvement and unique skin lesions such as cutaneous ulceration and palmar papules reflecting vasculopathy, commonly associated with rapidly progressive interstitial lung disease (RP-ILD), causing fatal outcomes ^(1)^. Spontaneous intramuscular hemorrhage (SIH) is an extremely rare but severe vasculopathy complication that remains under-recognized in patients with DM ^(2)^. Here, we report an unusual case of multiple SIH attributable to anti-MDA5 antibody-positive DM with RP-ILD.

## Case Report

**Figure 1.**
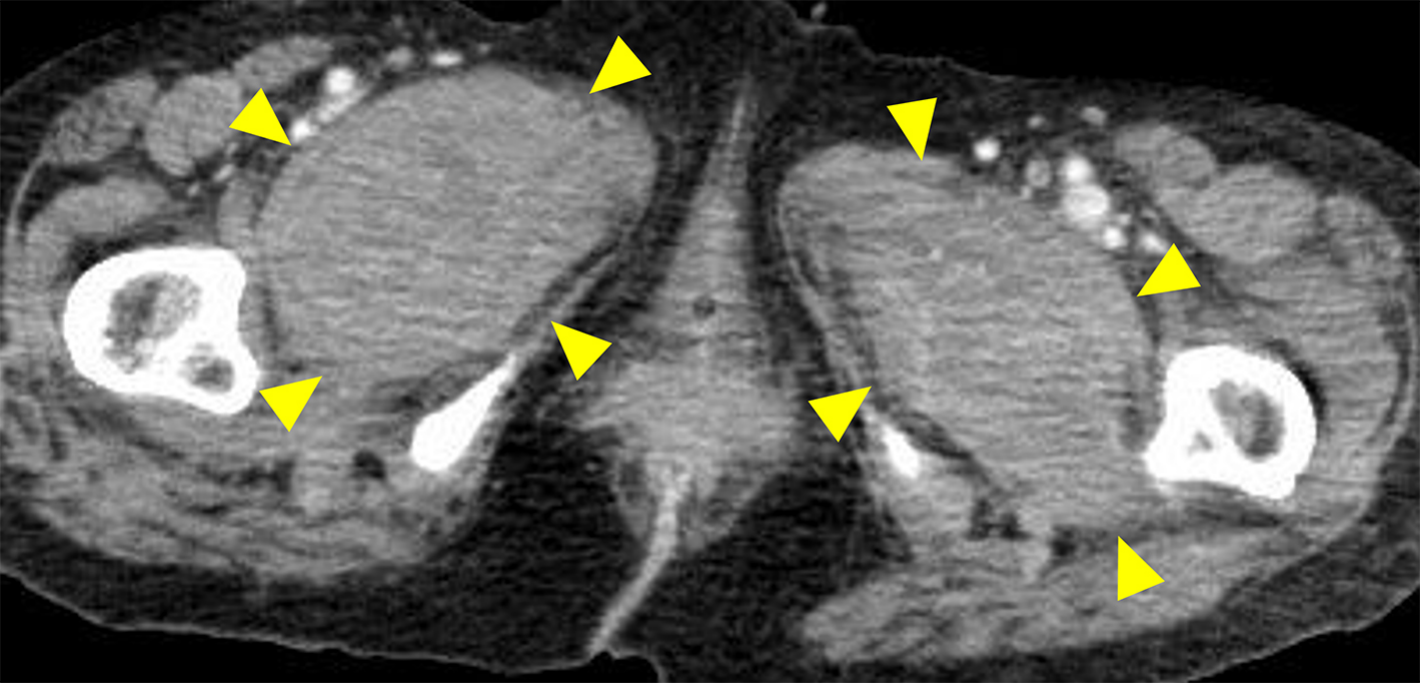
Contrast-enhanced computed tomography showing hemorrhages within bilateral adductor muscles (arrowheads).

**Figure 2.**
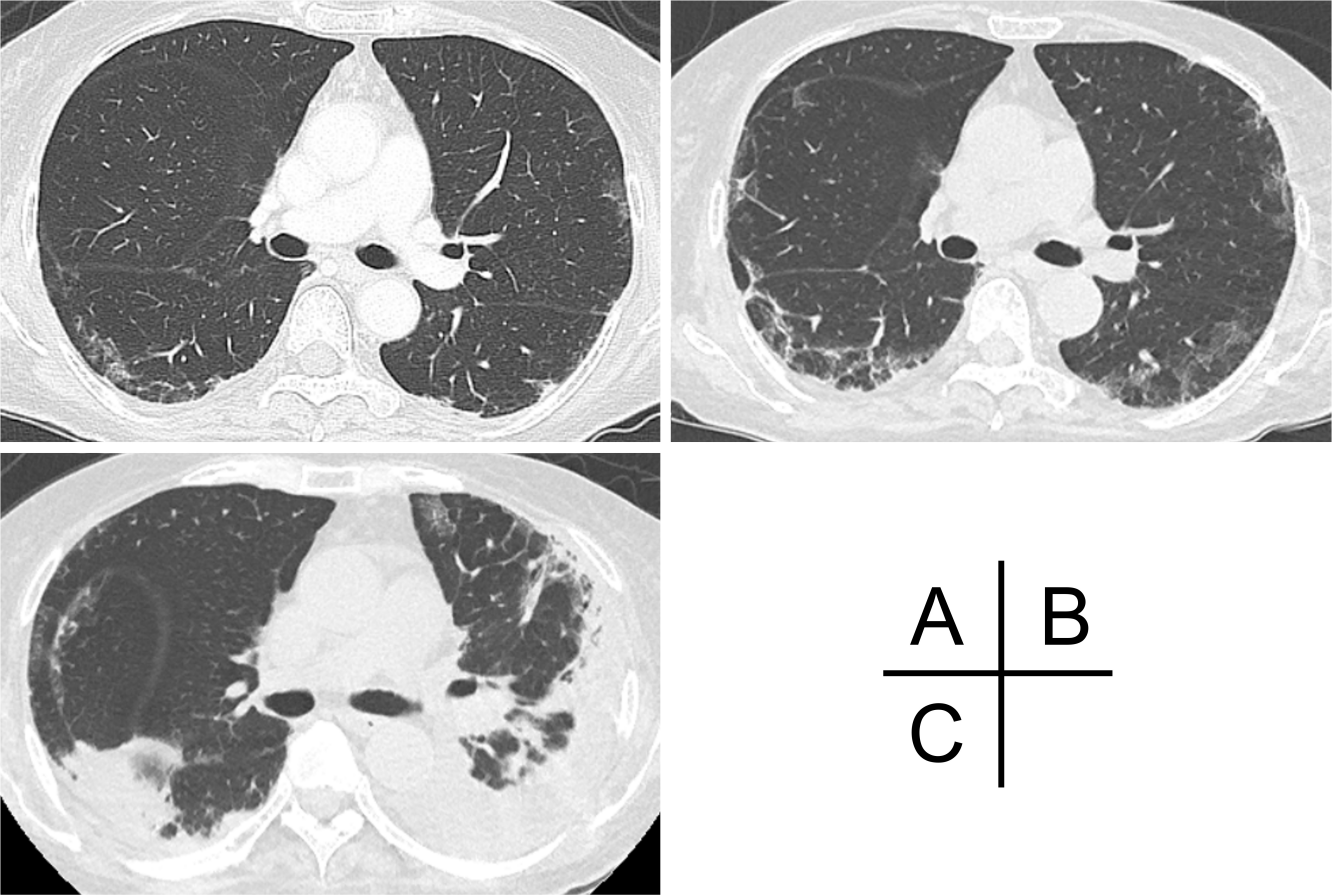
High-resolution computed tomography findings. (A) mild peripheral reticular shadows and GGOs in the bilateral lung lobes at the onset of spontaneous intramuscular hemorrhage. (B) Exacerbation of peripheral reticular shadows and GGOs in the bilateral lung lobes at the diagnosis of anti-MDA5 antibody-positive DM. (C) newly developed consolidation around the right interlobular pleura and increased pleural effusion in the left lung lobe along with worsening reticular shadows and GGOs in the bilateral lung lobes 1 month after remission induction therapy for anti-MDA5 antibody-positive DM. DM, dermatomyositis; GGO, ground-glass opacity; MDA5, melanoma differentiation-associated gene 5.

**Figure 3.**
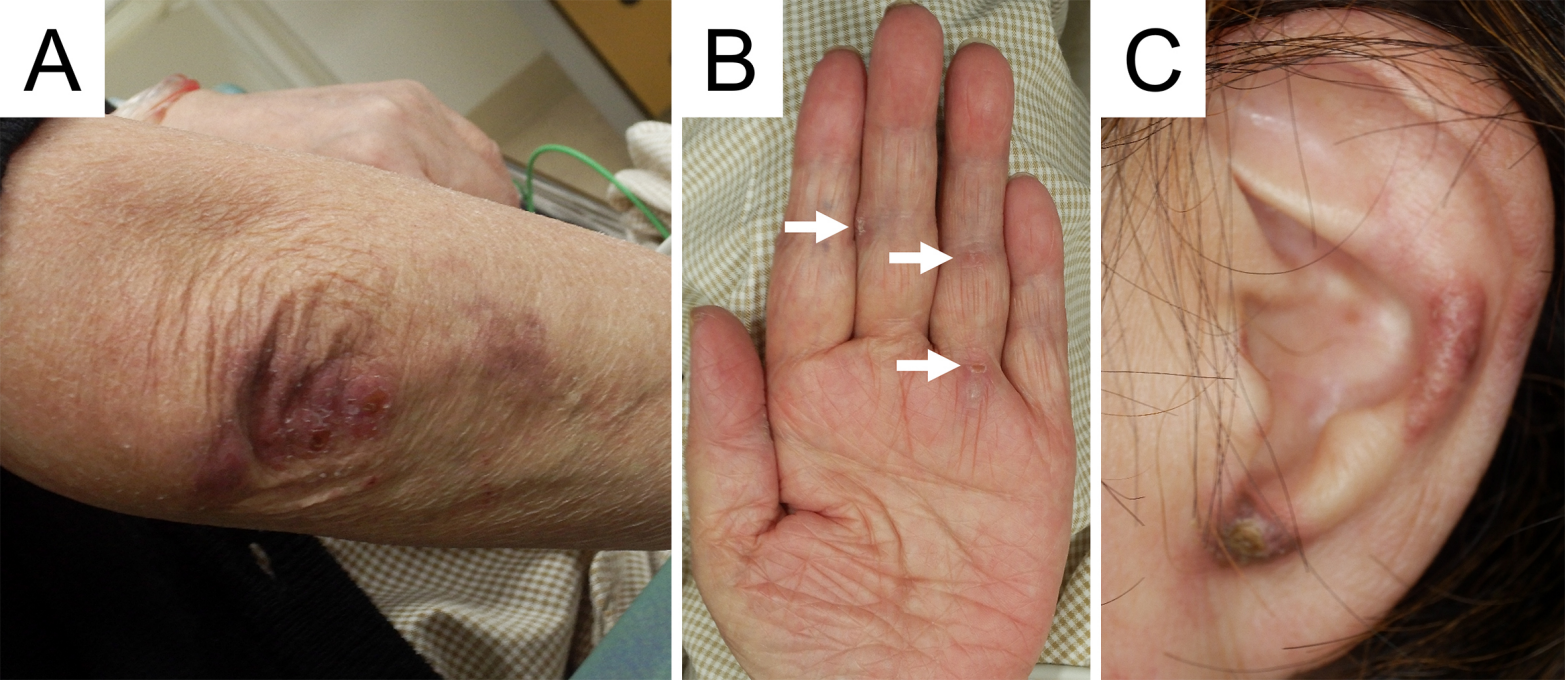
(A) Gottron’s sign on the extensor side of the left elbow. (B) Palmar papules (arrows). (C) Crusted purpura on helix and antihelix.

**Figure 4.**
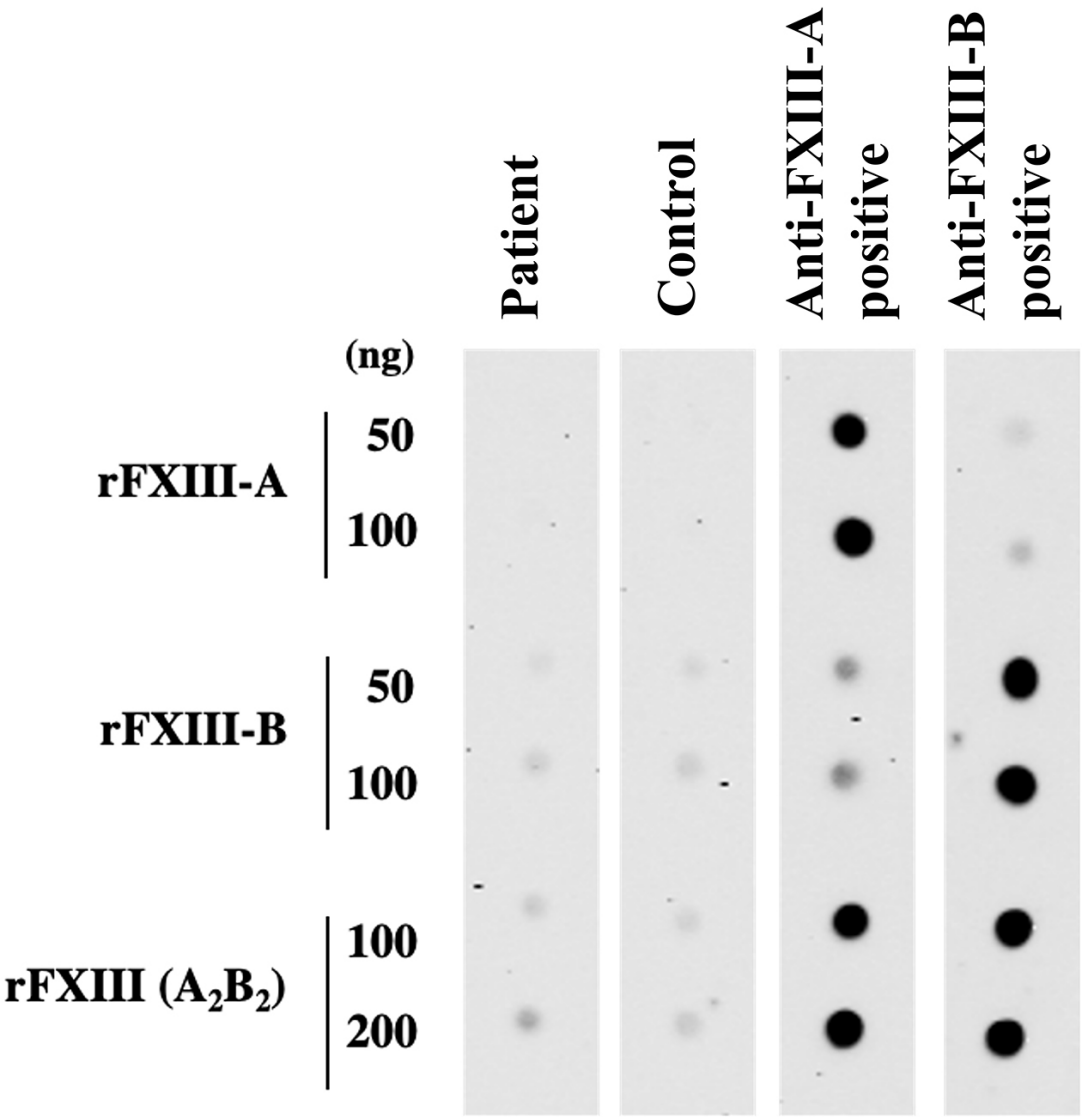
Immunoblotting test for detecting anti-factor XIII (FXIII) autoantibodies. Nitrocellulose membrane-bounded with recombinant FXIII-A subunit (rFXIII-A), recombinant FXIII-B subunit (rFXIII-B) proteins, and their complexes (rFXIIIA_2_B_2_) at the indicated amounts was incubated with diluted plasma obtained from healthy control, our patient, acquired FXIII deficiency patient with anti-FXIII-A autoantibody or anti-FXIII-B autoantibody. Immunoglobulin bound to rFXIII was then detected using a peroxidase-labelled anti-human immunoglobulin antibody.

A 72-year-old Japanese woman experienced fever, fatigue, and epigastric pain for 1 month with abnormal liver function including elevated levels of aspartate aminotransferase (427 U/L), alanine aminotransferase (127 U/L), and γ-glutamyl transferase (261 U/L) and positive anti-nuclear antibodies of a titer of 1:160 (speckled pattern), leading to a provisional diagnosis of autoimmune hepatitis. She was treated with moderate-dose prednisolone (PSL) for 3 weeks. However, acute-onset hematoma appeared in both her thighs, and she was transferred to our hospital for evaluation. No history of trauma, antithrombotic therapy, bleeding symptoms, or family history of bleeding was reported. On admission, physical examination revealed tender swelling with widespread purpura in both thighs without muscle weakness and respiratory symptoms. No typical cutaneous lesions indicative of DM, including a heliotrope rash, Gottron’s sign, mechanic’s hands, and palmar papules, were observed. Laboratory findings revealed elevated creatinine kinase (444 U/L) and ferritin levels (1834.9 ng/mL), with reduced hemoglobin (7.1 g/dL) and slightly low platelet count (131,000/μL). Contrast-enhanced computed tomography (CT) revealed hematoma within both adductor muscles (Figure 1), the right piriformis muscle, and right gluteus minimus without extravasation in addition to mild peripheral reticular shadows and ground-glass opacities (GGOs) in both lung lobes (Figure 2A). Coagulation study results were within the normal range except for decreased factor XIII (F13) activity (30%, reference: 70%-140%). Acquired F13 deficiency (AF13D) was considered based on the Japanese criteria for autoimmune AF13D ^(3)^, and the PSL dose was increased to 1 mg/kg/day with blood transfusions and F13 concentrate replacement, resulting in gradual hematoma resolution. The PSL dose was then tapered to 35 mg/day over 1 month; however, after 3 days, the patient experienced dyspnea on exertion and frequent nasal bleeding. Gottron’s signs on the elbows (Figure 3A), palmar papules (Figure 3B), and crusted purpura on the antihelix/helix (Figure 3C) were newly manifested. Skin biopsies revealed interfacial dermatitis consistent with DM. Exacerbation of peripheral reticular shadows and GGOs in bilateral lung lobes was observed on CT imaging (Figure 2B) with elevated Krebs von den Lungen-6 levels (734 U/mL). Anti-MDA5 antibody (3710 index, cut-off: <32 index) was clarified with commercially available enzyme-linked immunosorbent assay, but not anti-F13 autoantibodies by immunoblotting analysis (Figure 4). Anti-Ro52 antibody (268.3 index, cut-off: <10 index) was detected using a novel multiplex protein assay (A-Cube) ^(4)^. The final diagnosis was anti-MDA5 antibody-positive DM with RP-ILD. Furthermore, a re-analysis of stored serum samples collected at the onset of bilateral hematoma showed the presence of anti-MDA5 antibodies, supporting SIH due to anti-MDA5 antibody-positive DM. Remission induction therapy, including cyclophosphamide, tacrolimus, and tofacitinib was introduced, however, respiratory status gradually deteriorated. Newly developed consolidation around the right interlobular pleura and increased pleural effusion in the left lung lobe along with worsening reticular shadows and GGOs in the bilateral lung lobes were observed on CT imaging 1 month after remission induction therapy (Figure 2C). Eventually, she died of refractory RP-ILD-related respiratory failure 2 months after the treatments.

## Discussion

SIH typically occurs within 6 months, especially 1-2 months, after DM onset without a history of trauma, involving large arteries in the limbs, shoulders, abdomen, and iliolumbar regions ^(2), (5)^, and usually develops at multiple sites ^(6)^, as with this case. It most frequently presents as iliopsoas hematoma ^(2)^, typically resulting from hemophilia, antithrombotic therapy, traumatic injuries, rarely alcoholic liver cirrhosis, and hypertensive urgency ^(7)^. However, in the present case, clinical findings ruled out these conditions. The absence of anti-F13 autoantibodies finally ruled out autoimmune AF13D. In contrast, cutaneous lesions indicative of DM were not initially detected and manifested after the PSL dose was tapered, suggesting that moderate-dose PSL might have masked these manifestations. Therefore, the findings in this case underscore the significance of considering DM as a differential diagnosis when the etiology of SIH is unexplained. Especially in patients undergoing immunosuppressive therapy, physicians should always be vigilant for the development of cutaneous manifestations suggesting DM.

Although the underlying mechanisms of why SIH develops remain poorly understood, it is hypothesized that vessel-wall fragility due to endothelial damage caused by inflammatory processes in DM and excessive glucocorticoids-induced tissue fragility potentially results in abrupt hemorrhage, the likelihood of which prophylactic antithrombotic treatment increases ^(6), (8)^. As evidenced by extensive research, direct type I interferon-driven vasculopathy plays a central role in the prominent endothelial inflammation in DM, especially with anti-MDA5 antibody ^(9)^, which is the highest risk factor for SIH associated with DM ^(8)^. SIH developed more frequently early after the onset of DM in the majority of previously reported cases ^(2), (6)^, and massive bleeding occurred even after DM flares in one case ^(10)^, potentially indicating the link between the development of SIH and high disease activity of DM. Moreover, in this case, SIH developed 3 weeks after the initiation of moderate-dose PSL without antithrombotic drugs. Several DM patients had SIH even without anticoagulation ^(2)^, and high-dose PSL is commonly introduced to induce remission in severe autoimmune diseases. Taken together, the leading factor of DM-related SIH may be persistent immune-mediated vasculopathy.

Anti-MDA5 and Ro52 antibodies were significant determinants of developing SIH in DM ^(8)^. The mortality rate for DM-related SIH is 50%, with the rate exceeding 70% for anti-MDA5 and anti-Ro52 antibodies positivity, which is higher than that for anti-MDA5 or Ro52 antibody-single positive or negative SIH ^(6)^. Furthermore, anti-Ro52 antibody is most frequently observed in DM-related SIH ^(6), (8)^. Given the current limited evidence, further studies are warranted to clarify whether autoantibody profiles determine the prognosis even in DM-related SIH, or whether SIH further worsens the prognosis in DM. Nevertheless, evaluation of myositis-specific and myositis-associated autoantibodies is crucial to ensure adequate diagnosis when SIH associated with DM is highly suspected.

## Article Information

### Conflicts of Interest

None

### Acknowledgement

The authors would like to thank Editage (www.editage.jp) for English language editing, and Dr Masayaoshi Souri, Department of Molecular Patho-Biochemistry and Patho-Biology, Yamagata University School of Medicine, for measuring anti-factor XIII autoantibodies.

### Author Contributions

TM wrote the manuscript. HM and HF contributed to the critical review of the manuscript. All authors were involved in clinical management and approved the final manuscript.

### Informed Consent

Written informed consent for publication was obtained from the patient.
